# Supplementary material for: Continuous exchange of an inner-membrane ring component is required for assembly and function of the type III secretion system
Source: Nat Commun. 2025 Nov 10;16:9889. doi: 10.1038/s41467-025-65973-9 (PMC12603124; doi:10.1038/s41467-025-65973-9)
Supplement: Supplementary file 1 — Supplementary Information [file 41467_2025_65973_MOESM1_ESM.pdf]

Supplementary information for

Continuous exchange of the inner membrane ring component SctD is required for the assembly and function of the type III secretion system

Corentin Brianceau[1,2], Stephan Wimmi[1,#], Thales Kronenberger[3,4,5] & Andreas Diepold[1,2]

1: Max Planck Institute for Terrestrial Microbiology, Department of Ecophysiology, Marburg, Germany

2: Department of Applied Biology, Institute of Applied Biosciences, Karlsruhe Institute of Technology (KIT), Karlsruhe, Germany

3: German Center for Infection Research (DZIF), partner-site Tübingen, Tübingen, Germany

4: Institute of Medical Microbiology and Hygiene, Interfaculty Institute of Microbiology and Infection Medicine (IMIT), University of Tübingen, Tübingen, Germany

5: School of Pharmacy, Faculty of Health Sciences, University of Eastern Finland, Kuopio, Finland

#: current address: Institute for Biological Physics, University of Cologne, Cologne, Germany

Corresponding author:

Andreas Diepold, andreas.diepold@kit.edu, +49-721-608-45610

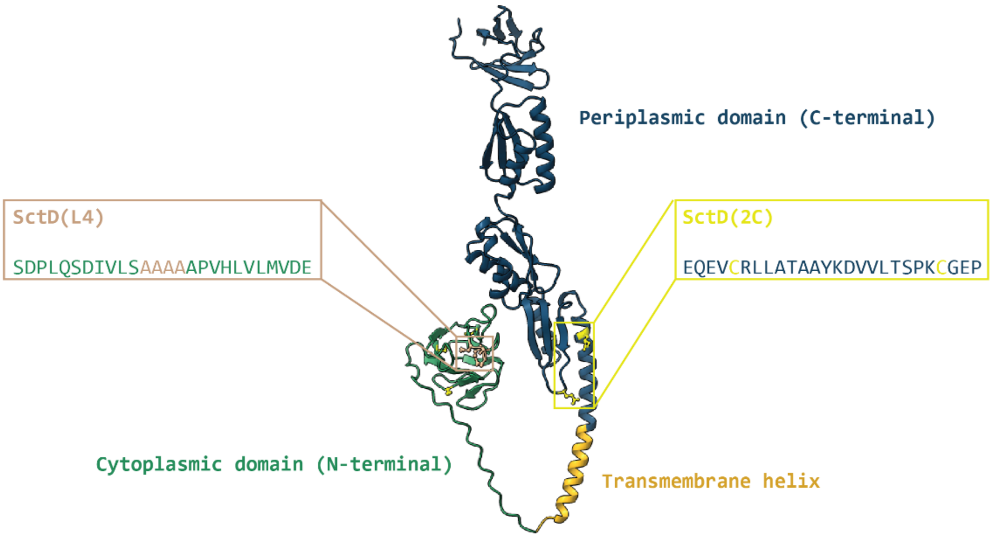


```
SctD(WT) 1   MSWVCRFYQGKHRGVEVELPHGRCVFGSDPLQSDIVLSDSEIAPVHLVLMVDEEGIRLTDSAEPLLQEGLPVPLGTLLRA 80
SctD(L4) 1   MSWVCRFYQGKHRGVEVELPHGRCVFGSDPLQSDIVLSAAAAAPVHLVLMVDEEGIRLTDSAEPLLQEGLPVPLGTLLRA 80
SctD(2C) 1   MSWVCRFYQGKHRGVEVELPHGRCVFGSDPLQSDIVLSDSEIAPVHLVLMVDEEGIRLTDSAEPLLQEGLPVPLGTLLRA 80

SctD(WT) 81  GTCLEVGFLLWTFVAVGQPLPETLQVPTQRKEPTDRLPRSRLGVGLGVLSLLLLLTFLGMLGHGLWREYNQDGQLVEQEV 160
SctD(L4) 81  GTCLEVGFLLWTFVAVGQPLPETLQVPTQRKEPTDRLPRSRLGVGLGVLSLLLLLTFLGMLGHGLWREYNQDGQLVEQEV 160
SctD(2C) 81  GTCLEVGFLLWTFVAVGQPLPETLQVPTQRKEPTDRLPRSRLGVGLGVLSLLLLLTFLGMLGHGLWREYNQDGQLVEQEV 160

SctD(WT) 161 RRLLATAAYKDVVLTSPKEGEPWLLTGYIQDNHARLSLQNFLESHGIPFRLELRSMEELRQGAEFILQRLGYHGIEVSLA 240
SctD(L4) 161 RRLLATAAYKDVVLTSPKEGEPWLLTGYIQDNHARLSLQNFLESHGIPFRLELRSMEELRQGAEFILQRLGYHGIEVSLA 240
SctD(2C) 161 CRLLATAAYKDVVLTSPKCGEPWLLTGYIQDNHARLSLQNFLESHGIPFRLELRSMEELRQGAEFILQRLGYHGIEVSLA 240

SctD(WT) 241 PQAGWLQLNGEVSEEIQKQKIDSLLQAEVPGLLGVENKVRIAGNQRKRLDALLEQFGLDSDFTVNVKGELIELRGQVNDE 320
SctD(L4) 241 PQAGWLQLNGEVSEEIQKQKIDSLLQAEVPGLLGVENKVRIAGNQRKRLDALLEQFGLDSDFTVNVKGELIELRGQVNDE 320
SctD(2C) 241 PQAGWLQLNGEVSEEIQKQKIDSLLQAEVPGLLGVENKVRIAGNQRKRLDALLEQFGLDSDFTVNVKGELIELRGQVNDE 320

SctD(WT) 321 KLSSFNQLQQTFRQEFGNRPKLELVNVGGQPQHDELNFEVQAISLGKVPYVVLDNHQRYPEGAILNNGVRILAIRRDAVI 400
SctD(L4) 321 KLSSFNQLQQTFRQEFGNRPKLELVNVGGQPQHDELNFEVQAISLGKVPYVVLDNHQRYPEGAILNNGVRILAIRRDAVI 400
SctD(2C) 321 KLSSFNQLQQTFRQEFGNRPKLELVNVGGQPQHDELNFEVQAISLGKVPYVVLDNHQRYPEGAILNNGVRILAIRRDAVI 400

SctD(WT) 401 VSKGKREFVIQLNGGKPR 418
SctD(L4) 401 VSKGKREFVIQLNGGKPR 418
SctD(2C) 401 VSKGKREFVIQLNGGKPR 418
```

Supplementary Figure 1 – Position of native and inserted cysteines and mutations in SctD(L4) and SctD(2C) variants in the SctD sequence and structure

Top, AlphaFold prediction of an SctD monomer with the cytoplasmic domain (N-term; green), transmembrane alpha helix (orange) and periplasmic domain (C-term; blue). Boxes depict the four Ala substitutions in the cytoplasmic domain for the mutant SctD(L4) and the two Cys substitutions in the periplasmic domain for the mutant SctD(2C). Bottom, sequence alignment of the indicated SctD variant. Green, cytosolic domain; orange, transmembrane domain; blue, periplasmic domain; yellow, cysteine residues; light salmon, alanine replacements in SctD(L4).

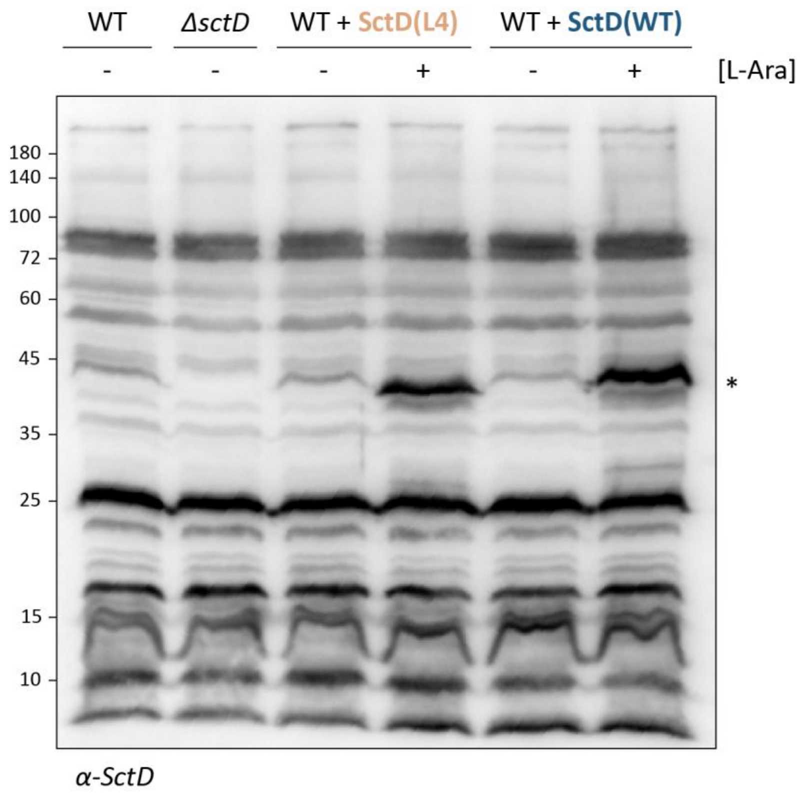

Supplementary Figure 2 – Expression of SctD variants from plasmid after native T3SS assembly

Expression of SctD from its native locus and overexpression of SctD(WT) and SctD(L4) from pBAD plasmids. The expression of SctD from the native locus was induced by incubation at 37°C. After assembly and during incubation at 28°C, expression of SctD variants was induced by addition of 0.01% L-Arabinose (+) or repressed by 0.2% L-Glucose (-). Immunoblot using antibodies directed against SctD. *: SctD (expected size: 46.8 kDa for WT, 46.7 kDa for L4 variant). Representative result, n = 3 independent experiments.

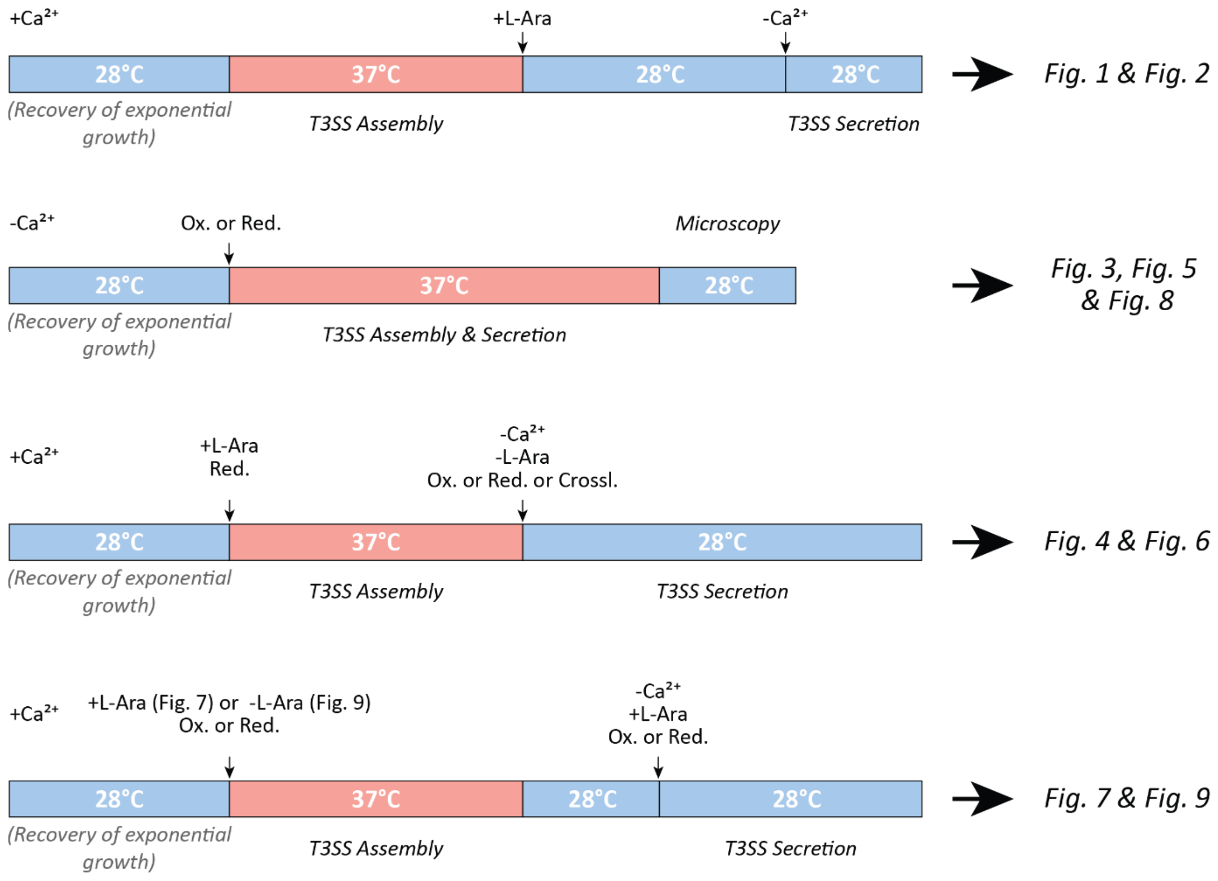

Code	Conditions	Effects
28°	Temperature of 28°	No T3SS expression
37°	Temperature of 37°	T3SS expression and assembly
$+Ca^{2+}$	5 mM $CaCl_2$	Inhibition of secretion by the T3SS
$-Ca^{2+}$	5 mM EGTA	Induction of secretion by the T3SS
Ox.	No DTT	Natural oxidative environment in the periplasm
Red.	2 mM DTT	Reducing environment in the periplasm
Cross.	0.1 mM $BM(PEG)_2$	Irreversible crosslinking of Cys residues
+L-Ara	L-Arabinose, as indicated	Expression of protein of interest from plasmid

Supplementary Figure 3 – Experimental setup and conditions to investigate SctD exchange and its role in *Yersinia*

Schematic representations of the different experimental conditions used to investigate the exchange of SctD and its role in the assembly and the function of the T3SS. The respective setups were used for the figures indicated on the right. Incubation at 37°C induces expression of the T3SS components from their native locus, while subsequent incubation at 28°C stops this expression. The cultures were grown either in non-secreting ($+Ca^{2+}$) or in secreting ($-Ca^{2+}$) conditions, with expression of the respective protein of interest from the pBAD plasmid induced (+L-Ara) or non-induced (-L-Ara) and with no treatment (Ox.), presence of DTT (Red.) or presence of $BM(PEG)_2$ (Crossl.), as indicated in the table at the bottom.

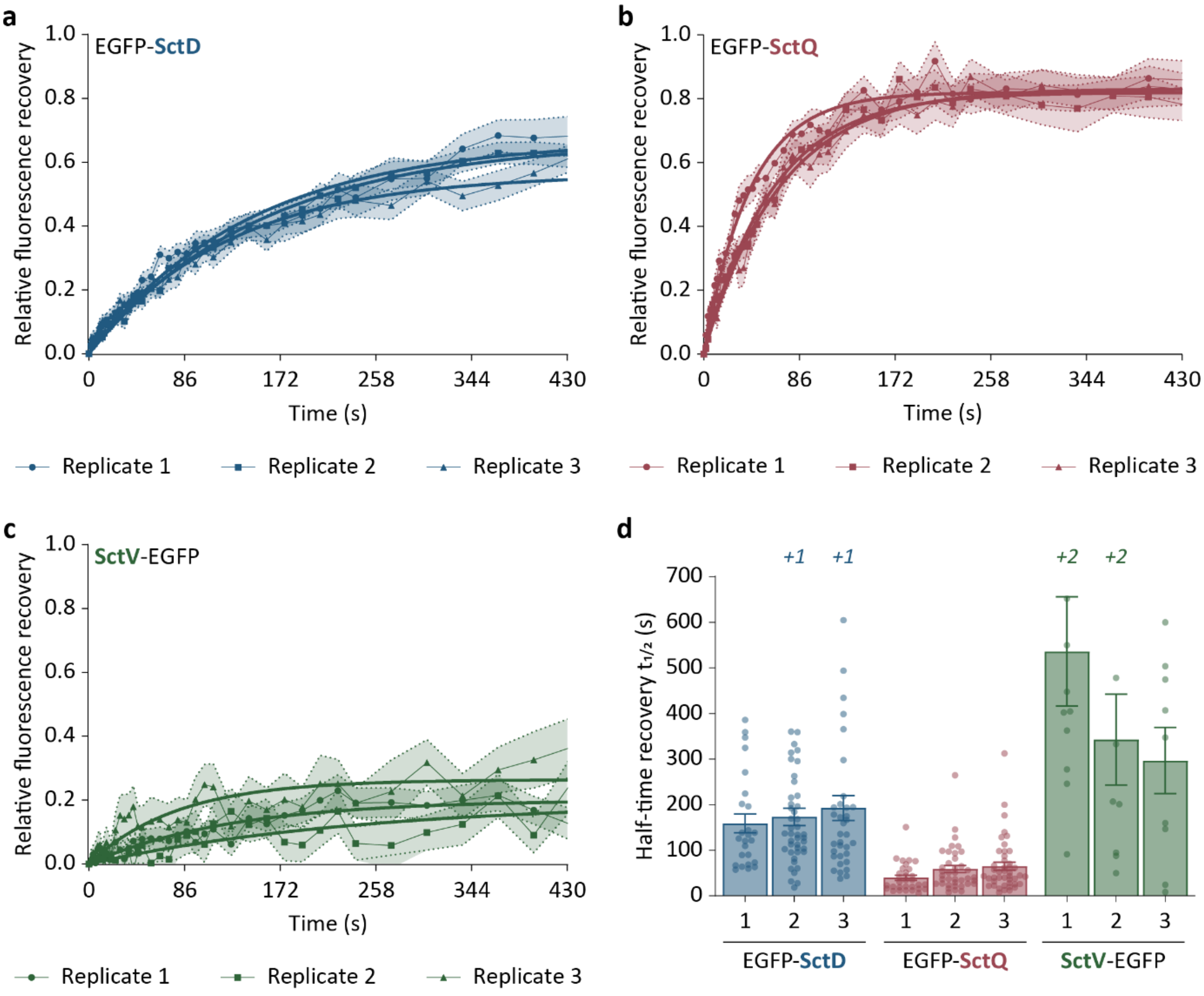

Supplementary Figure 4 – Reproducibility of the FRAP experiments for EGFP-SctD, EGFP-SctQ and SctV-EGFP

Average fluorescence recovery curves of each of three independent biological replicates for EGFP-SctD **(a)**, EGFP-SctQ **(b)**, and SctV-EGFP **(c)**. The fluorescence recovery curves represent the merged data from multiple foci with replicate 1 (circles), replicate 2 (squares) and replicate 3 (triangles). **d)** Average analysis of the half-time recoveries from the three replicates of each strain EGFP-SctD, EGFP-SctQ, and SctV-EGFP. The half-time recovery data were extracted from the fluorescence recovery curves with $R^2 > 0.4$ for exponential regression. The values below each column represent the replicate number for each protein, *+n*, additional values outside displayed y range (>700 s). From left to right, n = 25, 48, 35, 31, 38, 44, 11, 9, 9 individual recovery curves. Error bars represent the standard error of the mean.

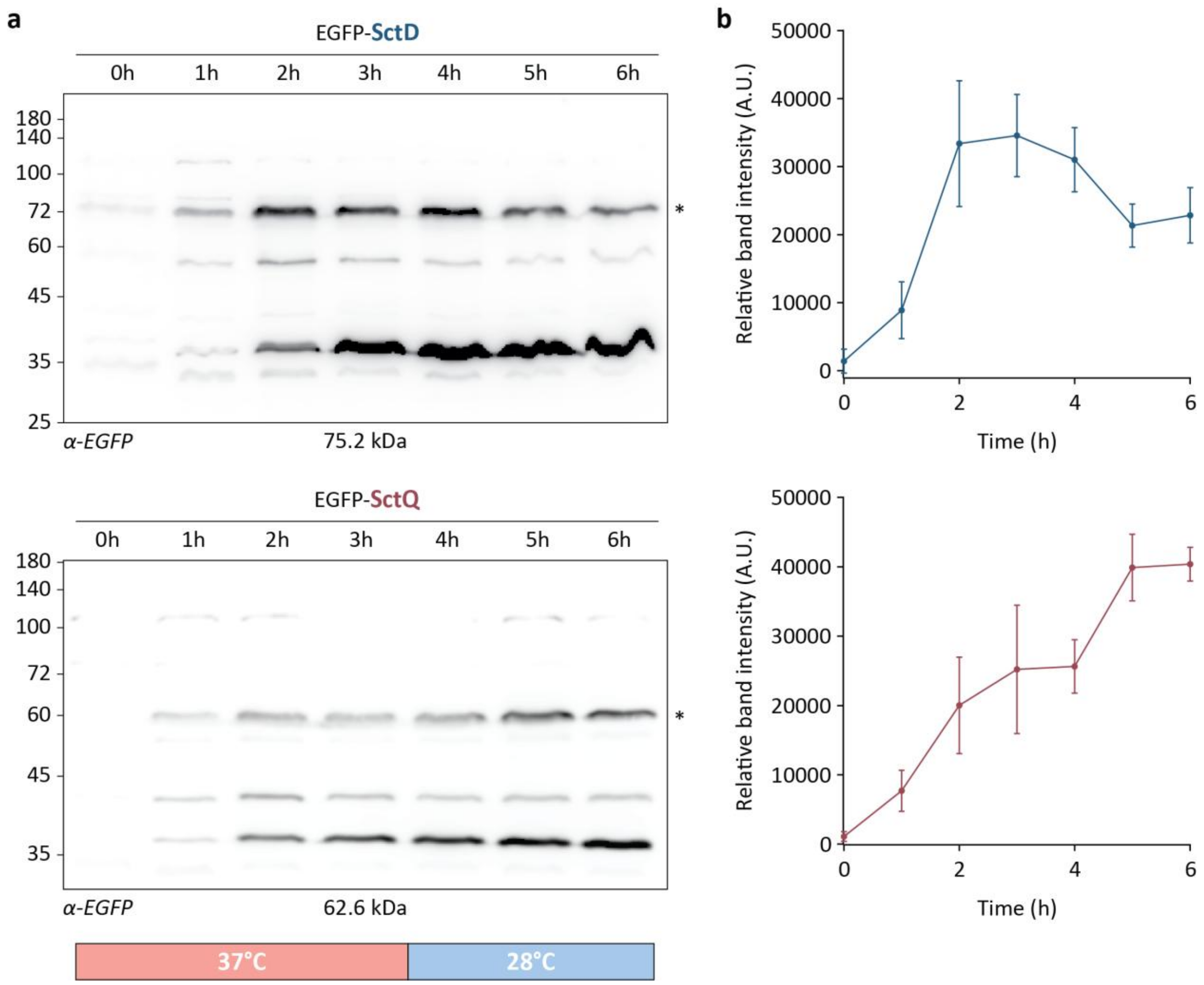

Supplementary Figure 5 - Cellular protein levels of the EGFP fusion proteins do not increase after temperature shift

a) Immunoblot time course with anti-EGFP of total cellular protein from 1.5 x 10^8 bacteria expressing EGFP-SctD or EGFP-SctQ from their native promoter on the pYV virulence plasmid under secretion conditions (with EGTA). The bacteria were incubated for 3 hours at 37°C (temperature inducing expression of T3SS components) and then shifted to 28°C for an additional 3 hours (temperature repressing expression of T3SS components). *: EGFP-SctD (blue; expected size: 75.2 kDa) or EGFP-SctQ (red; expected size: 62.6 kDa). Representative result, *n* = 3 independent experiments. **b)** Quantitative analysis of the cellular protein amount shown in (A). *n* = 3 independent experiments, error bars denote standard deviation.

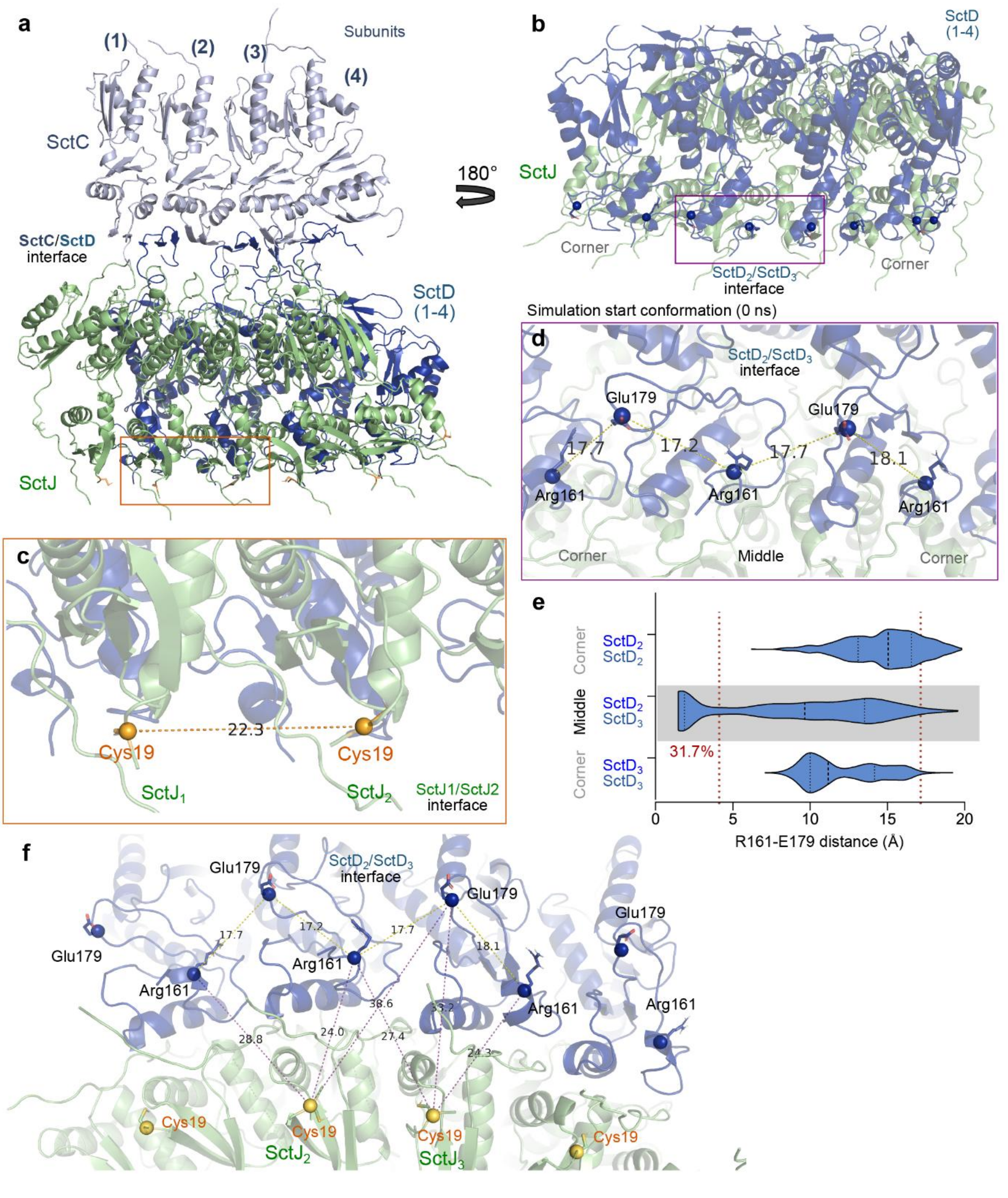

Supplementary Figure 6 – Structural modeling of *Yersinia enterocolitica* SctCDJ

a, b) Overview of the *Ye*SctCDJ model highlighting four repeated subunits containing one each: SctC (dark blue), SctD (turquoise) and SctJ (green) from the "front" (a) and a snippet of the "back" viewpoints (b), interfaces between SctC and SctD are labelled and interface between SctD subunits are highlighted by a square and further depicted in (c, d). **c, d)** Bottom part of the complex's interface in the initial model highlighting the SctJ's subunits, with the naturally occurring cysteine residues (c), or SctD's, highlighting the validated mutation sites, interfaces (d) and the respective distance between their alpha-carbons (depicted as spheres). Simulations were run for at least 1,500 ns (3x500 ns) and representative frames were retrieved from hierarchical clustering using the protein backbone position variation. SctD periplasmatic portion was modelled starting from Gln158 – Ala383. **e)** violin plot showing the distance variation between the side-chains of Arg161 and Glu179 along the simulation.

We calculated distances for residues within the “corner” protomers (SctD2 and SctD3) and of residues between SctD2 and SctD3 subunits (or “middle”), where they are below the 4.5 Å cut-off (dashed line) for ~32% of the simulation time. The second dashed line depicts the 17 Å distance observed in the initial models. **f)** Overview of the *Ye*SctCDJ model highlighting the distances between the indicated cysteine residues of SctD (blue) and SctJ (green) as in c) and d).

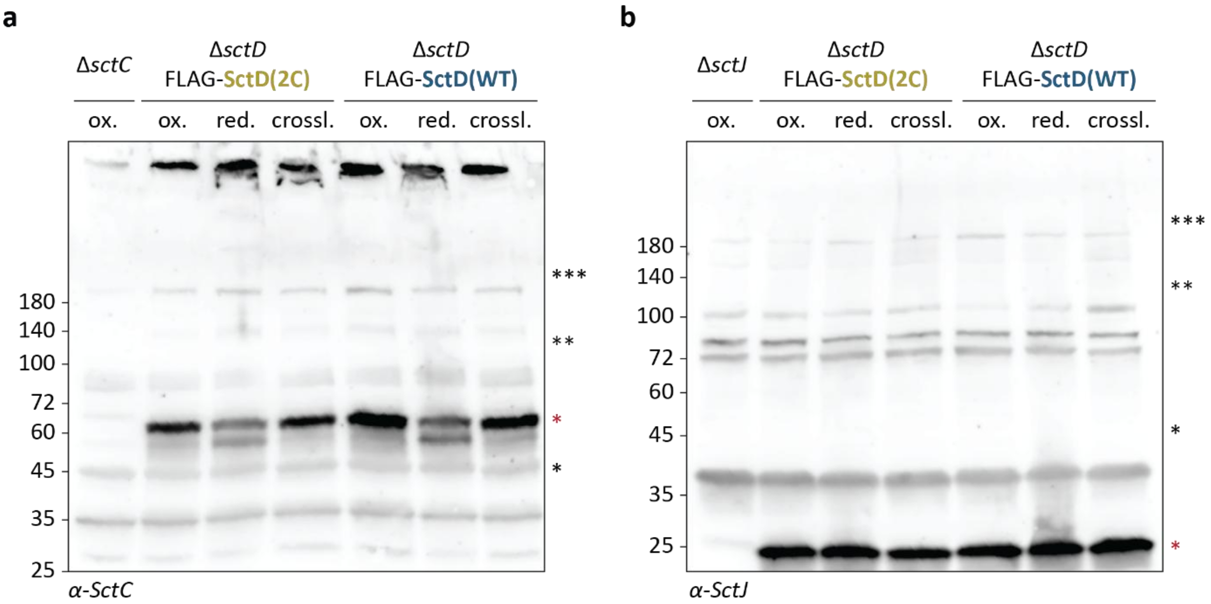

Supplementary Figure 7 – Absence of crosslinking of SctD(2C) with its periplasmic interacting partners SctC and SctJ

a, b) Western Blot anti-SctC (A) and anti-SctJ (B) of total cellular protein from 1.5 x 10^8 bacteria expressing either FLAG-SctD(2C) or FLAG-SctD(WT) from a plasmid in a Δ*sctD* background (n = 3 independent experiments). The expression of the different SctD variants was induced using 0.03% L-arabinose. The cultures were incubated at 28°C either untreated (ox.), in the presence of 2 mM of DTT (red.), or with 0.1 mM of BM(PEG)$_2$ (crossl.). To preserve the redox-sensitive crosslinks during the analysis, cells were resuspended in SDS sample buffer without DTT. Left, molecular weight in kDa; right, assignment of different expected crosslinking bands observed on the Western Blot in Fig. 4e, *: SctD monomer (45 kDa); **: Band running approximatively at 140 kDa molecular weight; ***: High molecular weight band; *: SctC monomer (a; 67.2 kDa) or SctJ monomer (b; 27.1 kDa).

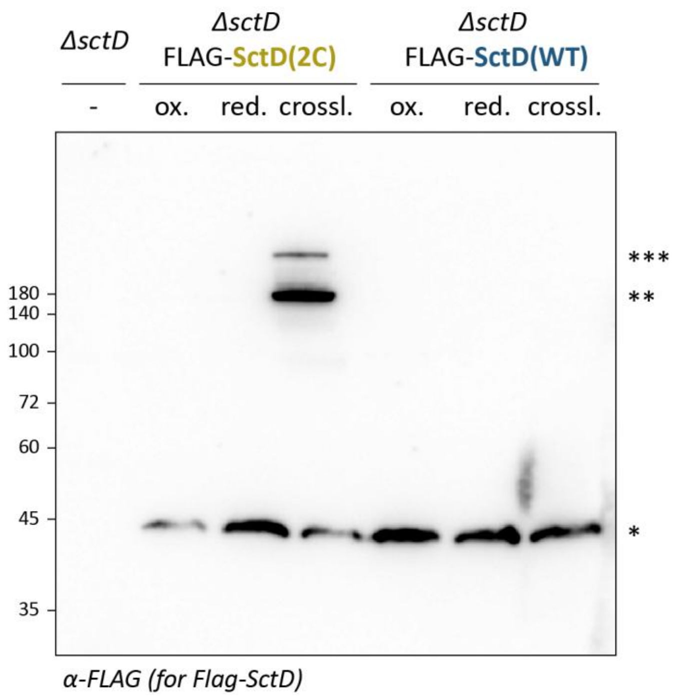

Supplementary Figure 8 – Analysis under reducing conditions specifically abolishes native oxidative SctD(2C) crosslinks

Western blot anti-FLAG of total cellular protein from 1.5 x 10^8 bacteria expressing either FLAG-SctD(2C) or FLAG-SctD(WT) from pBAD (*n* = 2 independent experiments). The expression of the different SctD variants was induced using 0.03% L-arabinose. The different cultures were untreated (ox.), or incubated with 2 mM of DTT (red.) or 0.1 mM of BM(PEG)$_2$ (crossl.). Each side of the SDS-PAGE represents the molecular weight in kDa (left) and the different bands observed on the Western Blot (right). *: SctD monomer (45 kDa); **: Band running approximatively at 140 kDa molecular weight; ***: High molecular weight band. Corresponds to non-reducing Western blot shown in Fig. 4d.

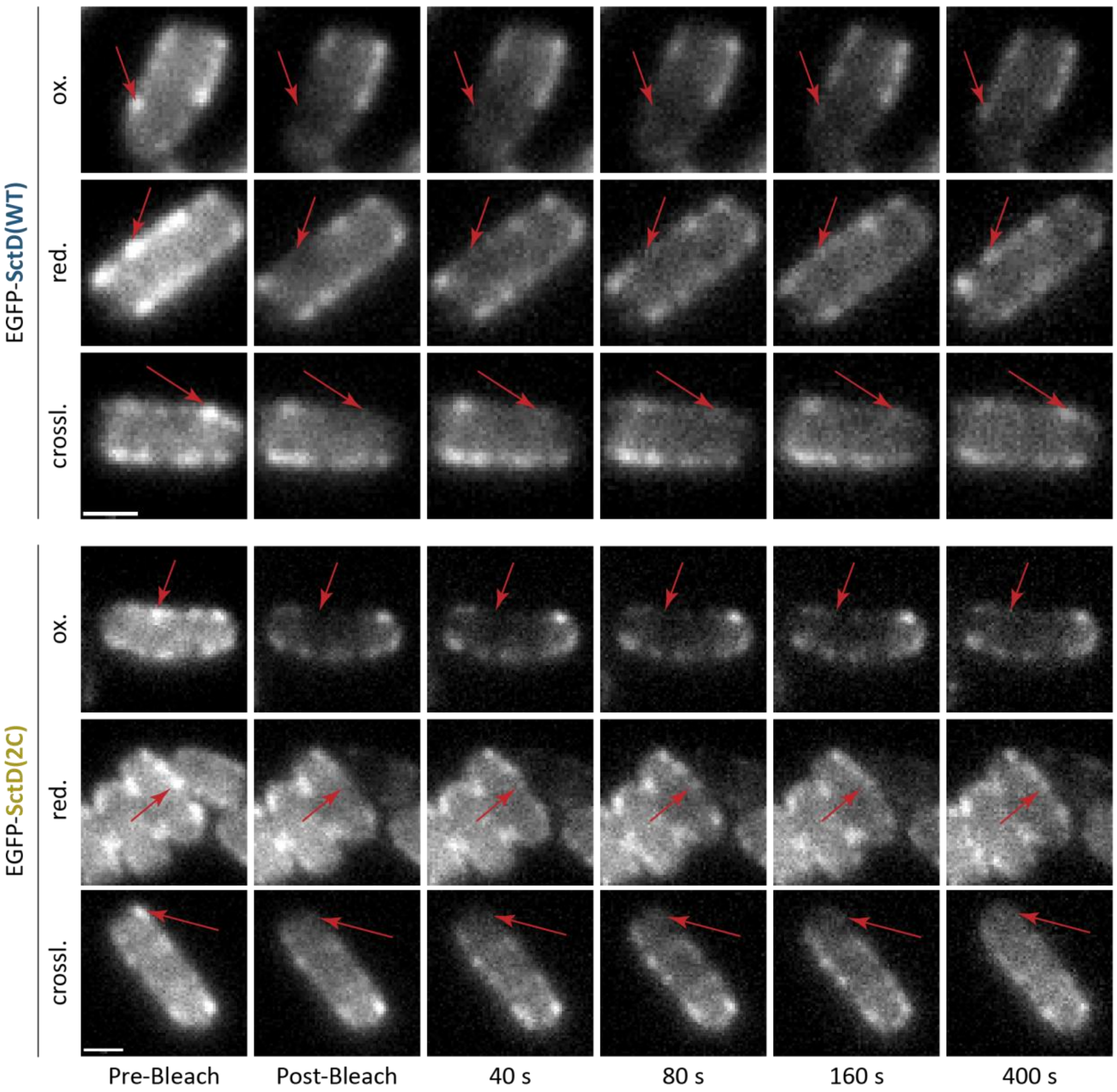

Supplementary Figure 9 – Fluorescence recovery of EGFP-SctD(WT) and SctD(2C) under conditions that modulate SctD(2C) crosslinking

Micrographs of cells expressing EGFP-SctD(WT) and EGFP-SctD(2C) under secreting conditions, before and after photobleaching, as indicated (*n* = 3 independent experiments). red./ox. = reducing/oxidizing environment (presence/absence of 2 mM DTT), crossl. = addition of 0.1 mM BM(PEG)$_2$. Red arrows indicate the photobleached spots. Scale bars represent 1 µm.

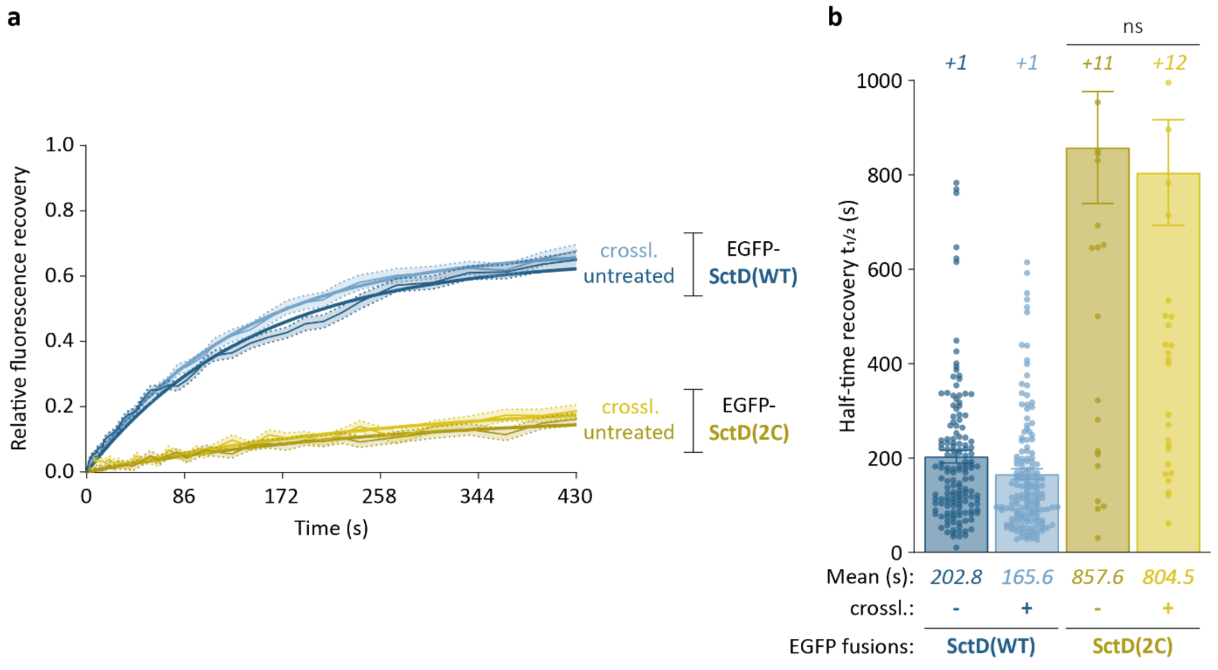

Supplementary Figure 10 – Crosslinking of SctD(2C) with BM(PEG)$_2$ does not affect SctD(2C) exchange more strongly than native oxidative crosslinking

a) Fluorescence recovery curves representing the data from multiple foci for the indicated strains in the absence (untreated) or presence (crossl.) of chemical crosslinker (0.1 mM BM(PEG)$_2$) under native oxidizing conditions (absence of DTT). Average fluorescence recovery curves (light connecting lines) are depicted with their standard errors of the mean (shaded areas) and exponential regression curves (thick lines). n = 179 for EGFP-SctD(WT) + BM(PEG)$_2$, 168 for EGFP-SctD(WT) untreated, 168 for EGFP-SctD(2C) + BM(PEG)$_2$, and 170 for EGFP-SctD(2C) untreated, from 3 independent experiments. **b)** Overall analysis of the half-time recoveries of the foci. The half-time recovery data for each strain were extracted from all individual fluorescence recovery curves with an R^2 value above 0.4. n = 148 individual recovery curves for EGFP-SctD(WT) untreated, 157 for EGFP-SctD(WT) + BM(PEG)$_2$, 29 for EGFP-SctD(2C) untreated, and 37 for EGFP-SctD(2C) + BM(PEG)$_2$). *+n*, additional values outside displayed y range (>1000 s). Error bars represent the standard error of the mean. ns, p = 0.7476 in a two-sided homoscedastic t test.

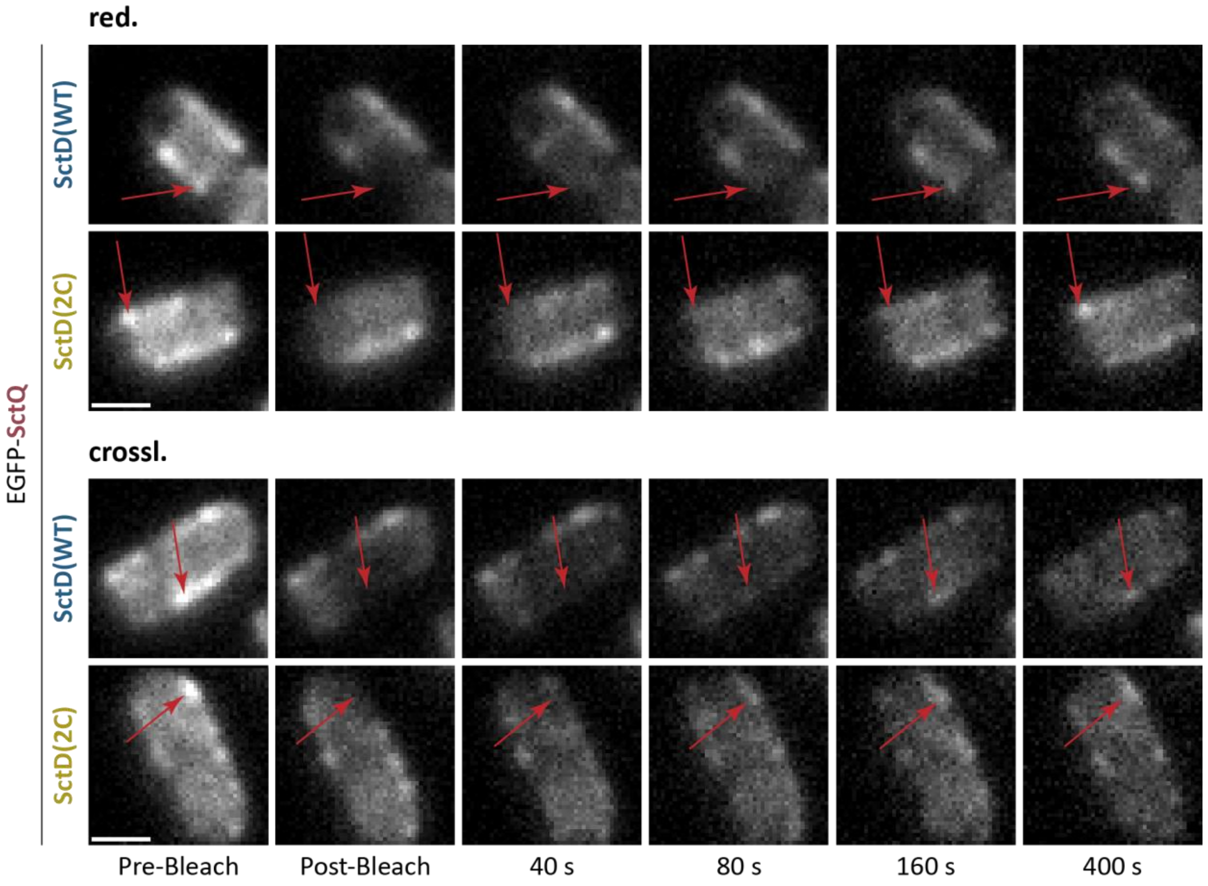

Supplementary Figure 11 – Fluorescence recovery of EGFP-SctQ in strains expressing SctD(WT) or SctD(2C) under conditions that modulate SctD(2C) crosslinking

Micrographs of cells expressing EGFP-SctQ in SctD(WT) or SctD(2C) under secreting conditions, before and after photobleaching, as indicated. n = 3 independent experiments. red. = reducing environment (presence of 2 mM DTT); crossl. = treatment with chemical crosslinker (0.1 mM BM(PEG)$_2$). Red arrows indicate the photobleached spots. Scale bars represent 1 µm.

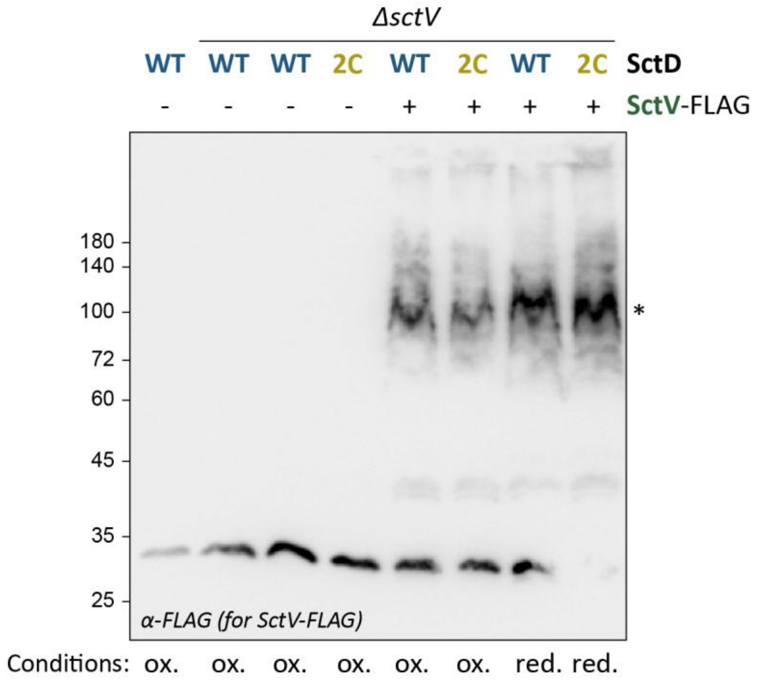

Supplementary Figure 12 – Induction of SctV expression from pBAD after completion of injectisome assembly

Representative Western Blot anti-FLAG of total cellular protein from 1.5 x 10^8 bacteria (*n* = 2 independent experiments). Expression of SctV-FLAG from pBAD was induced with 0.2% L-arabinose following the induction of T3SS component expression and assembly of the membrane rings. Left, molecular weight in kDa. *: SctV-FLAG monomer (76.4 kDa); unspecific background band at 30-35 kDa.

Suppl. Table 1 – Strains and plasmids used in this study

Strain	Genotype	Reference
MRS40	Wild-type *Y. enterocolitica* E40 *ΔblaA*	[1]
IML421*asd* (ΔHOPEMTasd)	MRS40 *yopH*$_{\Delta 1\text{-}352}$ *yopO*$_{\Delta 65\text{-}558}$ *yopP*$_{43}$ *yopE*$_{5}$ *yopM*$_{18}$ *yopT*$_{135}$ *Δasd*	[2]
AD4016	MRS40 *egfp-sctQ*	[3]
AD4037	MRS40 *ΔsctV*	[3]
AD4050	MRS40 *egfp-sctD*	This study
AD4051	MRS40 *ΔsctD*	[3]
AD4052	MRS40 *egfp-sctQ*; *ΔsctD*	This study
AD4085	IML421*asd egfp-sctQ*	[2]
AD4175	IML421*asd sctV-egfp*	[4]
AD4306	IML421*asd egfp-sctD*	[5]
CB4004	IML421*asd egfp-sctD(2C)*	This study
CB4009	MRS40 *sctD(2C)*	This study
CB4010	MRS40 *ΔsctV*; *sctD(2C)*	This study
CB4011	MRS40 *egfp-sctQ*; *sctD(2C)*	This study
CB4017	MRS40 *ΔsctS*; *sctD(2C)*	This study

Plasmid	Genotype	Reference
pBAD-His B	pBR322-derived expression vector	Invitrogen
pKNG101	*oriR6K sacBR*$^{+}$ *oriTRK2 strAB*$^{+}$ (suicide vector for homologous recombination)	[6]
pAD153	pBAD::*sctV*	[7]
pAD321	pKNG101-*egfp-sctQ* (mutator for integration of *egfp*)	This study
pAD603	pBAD::*sctV-flag*	[4]
pAD780	pBAD::*yopE-flag*	This study
pAD793	pKNG101-*sctD(2C)* (mutator for replacement of R161C; E179C)	This study
pCB005	pBAD::*sctD(2C)*	This study
pCB025	pBAD::*flag-sctD(2C)*	This study
pCB039	pBAD::*flag-sctD*	This study
pCB046	pBAD::*sctD(L4)* (D39R; S40R; E41E; I42R)	This study
pCB075	pBAD::*sctS*	This study
pSW022	pBAD::*sctD*	[4]

Suppl. Table 2 – Oligonucleotides used in this study

Primer name	Sequence (5´→ 3´)	Used for
AD1445	TATAGGTCTCGGGCCCGTTGGGGACTCTTCTTCGCG	pAD793
AD1446	TATAGGTCTCTCTAGATTCAGCTTGCAGCAGGCTAT	pAD793
AD1447	GTCGTTTTAACATCGCCCAAATGT (Sequencing)	pAD793
AD1269	GCAAGAAGTATGTCGCTTGCTGG (Substitution R161C)	pCB005
AD1270	TCAACAAGTTGTCCATCC (Substitution R161C)	pCB005
AD1271	ATCGCCCAAATGTGGTGAACCTTG (Substitution E179C)	pCB005
AD1272	GTTAAAACGACATCCTTG (Substitution E179C)	pCB005
AD1384	GACTTCATGAGTGACTACAAGGACGACGATGATAAGGGTGGAGCAGGTGGTGCC GGAGGTAGTTGGGTCTGTCGTTTTTATCA (Integration FLAG-Tag)	pCB025
AD798	GACTAGATCTTCATCGAGGTTTACCTCCATTG (Integration FLAG-Tag)	pCB025
AD1384	GACTTCATGAGTGACTACAAGGACGACGATGATAAGGGTGGAGCAGGTGGTGCC GGAGGTAGTTGGGTCTGTCGTTTTTATCA (Integration FLAG-Tag)	pCB039
AD798	GACTAGATCTTCATCGAGGTTTACCTCCATTG (Integration FLAG-Tag)	pCB039
AD1725	GCAGCAGCACCCGTGCATTTAGTG	pCB046
AD1726	GGCGGCAGAAAGAACAATATCTGATTGCAAC	pCB046
AD2356	TATATCATGAGTCAAGGTGACATAATTCACTTCACCAGTCAGGCAT	pCB075
AD2358	TATAGAATTCTCATCTTATGCCTTGTATCTTCATCATGGTCATTTC	pCB075

Supplementary References:

1. Sory, M.-P., Boland, A., Lambermont, I. & Cornelis, G. R. Identification of the YopE and YopH domains required for secretion and internalization into the cytosol of macrophages, using the cyaA gene fusion approach. *Proc. Natl. Acad. Sci. U. S. A.* **92**, 11998–12002 (1995).
2. Kudryashev, M. *et al.* In situ structural analysis of the Yersinia enterocolitica injectisome. *Elife* **2**, e00792 (2013).
3. Diepold, A. *et al.* Deciphering the assembly of the Yersinia type III secretion injectisome. *EMBO J.* **29**, 1928–40 (2010).
4. Wimmi, S. *et al.* Dynamic relocalization of cytosolic type III secretion system components prevents premature protein secretion at low external pH. *Nat. Commun.* **12**, 1625 (2021).
5. Diepold, A., Kudryashev, M., Delalez, N. J., Berry, R. M. & Armitage, J. P. Composition, Formation, and Regulation of the Cytosolic C-ring, a Dynamic Component of the Type III Secretion Injectisome. *PLOS Biol.* **13**, e1002039 (2015).
6. Kaniga, K., Delor, I. & Cornelis, G. R. A wide-host-range suicide vector for improving reverse genetics in Gram-negative bacteria: inactivation of the blaA gene of Yersinia enterocolitica. *Gene* **109**, 137–41 (1991).
7. Diepold, A., Wiesand, U. & Cornelis, G. R. The assembly of the export apparatus (YscR,S,T,U,V) of the Yersinia type III secretion apparatus occurs independently of other structural components and involves the formation of an YscV oligomer. *Mol. Microbiol.* **82**, 502–14 (2011).
