# Supplementary material for: Continuous exchange of an inner-membrane ring component is required for assembly and function of the type III secretion system
Source: Nat Commun. 2025 Nov 10;16:9889. doi: 10.1038/s41467-025-65973-9 (PMC12603124; doi:10.1038/s41467-025-65973-9)
Supplement: Supplementary file 2 — Reporting Summary [file 41467_2025_65973_MOESM2_ESM.pdf]

nature portfolio

Corresponding author(s): Andreas Diepold

Last updated by author(s): Oct 14, 2025

Reporting Summary

Nature Portfolio wishes to improve the reproducibility of the work that we publish. This form provides structure for consistency and transparency in reporting. For further information on Nature Portfolio policies, see our Editorial Policies and the Editorial Policy Checklist.

Statistics

For all statistical analyses, confirm that the following items are present in the figure legend, table legend, main text, or Methods section.

n/a	Confirmed	
☐	☒	The exact sample size (n) for each experimental group/condition, given as a discrete number and unit of measurement
☐	☒	A statement on whether measurements were taken from distinct samples or whether the same sample was measured repeatedly
☐	☒	The statistical test(s) used AND whether they are one- or two-sided<br>*Only common tests should be described solely by name; describe more complex techniques in the Methods section.*
☒	☐	A description of all covariates tested
☒	☐	A description of any assumptions or corrections, such as tests of normality and adjustment for multiple comparisons
☐	☒	A full description of the statistical parameters including central tendency (e.g. means) or other basic estimates (e.g. regression coefficient) AND variation (e.g. standard deviation) or associated estimates of uncertainty (e.g. confidence intervals)
☐	☒	For null hypothesis testing, the test statistic (e.g. F, t, r) with confidence intervals, effect sizes, degrees of freedom and P value noted<br>*Give P values as exact values whenever suitable.*
☒	☐	For Bayesian analysis, information on the choice of priors and Markov chain Monte Carlo settings
☒	☐	For hierarchical and complex designs, identification of the appropriate level for tests and full reporting of outcomes
☒	☐	Estimates of effect sizes (e.g. Cohen's d, Pearson's r), indicating how they were calculated

Our web collection on statistics for biologists contains articles on many of the points above.

Software and code

Policy information about availability of computer code

Data collection	Micrographs were collected using using softWoRx 7.0.0
Data analysis	Image processing, fluorescence quantification, and band intensity measurements in both stained SDS-PAGE gels and immunoblots by gel densitometry was performed using the Fiji software (ImageJ 1.51f/1.52i/1.52n, https://fiji.sc/). The data were plotted using GraphPad Prism version 10.1.0 for Windows (GraphPad Software, Boston, Massachusetts USA). For the molecular modelling, the homology model was executed with Prime 2021.3 (Schrödinger LLC), which was followed by Protein Preparation Wizard 2021.3 (Schrödinger LLC). Maestro simulation interaction analysis tool (Schrödinger LLC) was used for the analysis of RMSD and interaction analysis.<br>To quantify the number of fluorescent foci, individual Y. enterocolitica cells were segmented using the StarDist neural network. The StarDist model was trained on the ZeroCostDL4Mic platform. The model is available in the DeepBacs collection on Zenodo (doi: 10.5281/zenodo.11105050). Foci localization was performed using the ThunderSTORM plugin in the Fiji software.

For manuscripts utilizing custom algorithms or software that are central to the research but not yet described in published literature, software must be made available to editors and reviewers. We strongly encourage code deposition in a community repository (e.g. GitHub). See the Nature Portfolio guidelines for submitting code & software for further information.

Data

Policy information about availability of data

All manuscripts must include a data availability statement. This statement should provide the following information, where applicable:

- Accession codes, unique identifiers, or web links for publicly available datasets
- A description of any restrictions on data availability
- For clinical datasets or third party data, please ensure that the statement adheres to our policy

Raw data trajectories, relevant conformations and interaction data of the molecular modelling of SctCDJ are available on Zenodo (doi: 10.5281/zenodo.13134138). The dataset and the model used for the quantification of the foci using DeepBacs are available in the DeepBacs collection on Zenodo (doi: 10.5281/zenodo.11105050). All other relevant data are included in the paper and/or its supplementary information files. Source data are provided with this paper.

Research involving human participants, their data, or biological material

Policy information about studies with human participants or human data. See also policy information about sex, gender (identity/presentation), and sexual orientation and race, ethnicity and racism.

Reporting on sex and gender	Not applicable for this study.
Reporting on race, ethnicity, or other socially relevant groupings	Not applicable for this study.
Population characteristics	Not applicable for this study.
Recruitment	Not applicable for this study.
Ethics oversight	Not applicable for this study.

Note that full information on the approval of the study protocol must also be provided in the manuscript.

Field-specific reporting

Please select the one below that is the best fit for your research. If you are not sure, read the appropriate sections before making your selection.

[x] Life sciences [ ] Behavioural & social sciences [ ] Ecological, evolutionary & environmental sciences

For a reference copy of the document with all sections, see nature.com/documents/nr-reporting-summary-flat.pdf

Life sciences study design

All studies must disclose on these points even when the disclosure is negative.

Sample size	No preliminary sample-size calculation was determined. Samples sizes were chosen to allow a clear test of the conclusions drawn from the presented data based on similar experiments, or as n>=3 where no such previous information was available. As the variance across experiments was low in most cases, no explicit power calculations were considered necessary.
Data exclusions	No data were excluded.
Replication	Experiments were performed in independent replicates as indicated in the respective figure legends, material and methods, and/or the Supplementary Information.
Randomization	Random allocation with regard to covariate is not applicable to the experiments carried out; no allocation into experimental groups was performed.
Blinding	Blinding was not required as the data processing did not rely on subjective assignments.

Reporting for specific materials, systems and methods

We require information from authors about some types of materials, experimental systems and methods used in many studies. Here, indicate whether each material, system or method listed is relevant to your study. If you are not sure if a list item applies to your research, read the appropriate section before selecting a response.

Materials & experimental systems

n/a	Involved in the study
☐	☒ Antibodies
☒	☐ Eukaryotic cell lines
☒	☐ Palaeontology and archaeology
☒	☐ Animals and other organisms
☒	☐ Clinical data
☒	☐ Dual use research of concern
☒	☐ Plants

Methods

n/a	Involved in the study
☒	☐ ChIP-seq
☒	☐ Flow cytometry
☒	☐ MRI-based neuroimaging

Antibodies

Antibodies used

Primary rabbit antibodies against SctC (MIPA250, 1:1,000), SctD (MIPA232, 1:1,000), SctJ (MIPA66, 1:5000), or the FLAG peptide (Rockland 600-401-383, 1:2,000) were used, followed by a secondary anti-rabbit antibody conjugated to horseradish peroxidase (Sigma-Aldrich A8275 1:10,000).

Validation

Validation statements and examples on manufacturers' sites: https://www.sigmaaldrich.com/GB/en/product/sigma/a8275 and in Diepold et al., EMBO J (2010), doi: 10.1038/emboj.2010.84. Additional validation by controls used in respective experiments.

Plants

Seed stocks

Report on the source of all seed stocks or other plant material used. If applicable, state the seed stock centre and catalogue number. If plant specimens were collected from the field, describe the collection location, date and sampling procedures.

Novel plant genotypes

Describe the methods by which all novel plant genotypes were produced. This includes those generated by transgenic approaches, gene editing, chemical/radiation-based mutagenesis and hybridization. For transgenic lines, describe the transformation method, the number of independent lines analyzed and the generation upon which experiments were performed. For gene-edited lines, describe the editor used, the endogenous sequence targeted for editing, the targeting guide RNA sequence (if applicable) and how the editor was applied.

Authentication

Describe any authentication procedures for each seed stock used or novel genotype generated. Describe any experiments used to assess the effect of a mutation and, where applicable, how potential secondary effects (e.g. second site T-DNA insertions, mosiacism, off-target gene editing) were examined.
